# Self-Micellizing Technology Improves the Properties of Ezetimibe and Increases Its Effect on Hyperlipidemic Rats

**DOI:** 10.3390/pharmaceutics11120647

**Published:** 2019-12-03

**Authors:** Carlos Torrado-Salmerón, Víctor Guarnizo-Herrero, Javier Cerezo-Garreta, Guillermo Torrado Durán, Santiago Torrado-Santiago

**Affiliations:** 1 Department of Pharmaceutics and Food Technology, Faculty of Pharmacy, Complutense University, Plaza Ramón y Cajal s/n, 28040 Madrid, Spain; ctorrado@ucm.es (C.T.-S.); victor08@ucm.es (V.G.-H.); jcerez01@ucm.es (J.C.-G.); 2 Department of Biomedical Sciences, Faculty of Pharmacy, University of Alcalá de Henares, Ctra Madrid-Barcelona Km 33,600, 28805 Madrid, Spain; guillermo.torrado@uah.es; 3 Instituto Universitario de Farmacia Industrial, Complutense University, Plaza Ramón y Cajal s/n, 28040 Madrid, Spain

**Keywords:** ezetimibe, solid dispersion, micellar systems, antihyperlipidemic activity, liver steatosis

## Abstract

The aim of this work was to develop ezetimibe self-micellizing solid dispersions using Kolliphor^®^ RH40 (MS-K) as a surfactant incorporating ezetimibe (EZ) into the croscarmellose hydrophilic carrier. Different ezetimibe:Kolliphor^®^ ratios were studied to select micellar systems that improve the dissolution properties of ezetimibe. The different formulations were characterized by means of solid state analysis by SEM, powder X-ray diffraction (PXRD), differential scanning calorimetry (DSC), and dissolution studies. These physicochemical studies showed a decrease from the crystalline structure of ezetimibe (EZ) to its amorphous state in the micellar systems (MS-K). A rapid dissolution profile was observed in these micellar systems compared to the drug raw material and physical mixture. Efficacy studies were conducted using a high-fat diet that induced hyperlipidemic rats. The micellar system selected (MS-K 1:0.75) revealed a significant improvement in serum levels of total cholesterol (TC), low-density lipoproteins (LDL), and triglycerides (TG) compared to ezetimibe raw material. The histopathological examination of liver tissue also showed that this micellar system exhibited more beneficial effects on liver steatosis compared to ezetimibe raw material (EZ-RM) and the high-fat diet group (HFD). This study suggests that EZ micellar systems using Kolliphor^®^ RH40 could enhance the antihyperlipidemic effect of ezetimibe and reduce liver steatosis.

## 1. Introduction

Hyperlipidemia is characterized by an increase in cholesterol, triglyceride, and lipoprotein levels [1,2]. The World Health Organization considers that hyperlipidemia has become more relevant in recent years because it is significantly associated with almost half the global cases of ischemic heart disease [2]. Hyperlipidemia induces hardening of the arteries and decreases blood flow to the aortic valve, this is an important risk factor for coronary artery disease (CAD). Several studies have shown an association between CAD and non-alcoholic fatty liver disease (NAFLD) [3,4]. NAFLD disorders commonly feature an accumulation of excessive fat in the liver, in which non-alcoholic steatohepatitis, ballooning, lobular inflammation, and other cell injuries are observed [1,5].

Ezetimibe (EZ) is an antihyperlipidemic agent with fewer adverse side effects than statins, and it is usually used in combination with them for the treatment of hyperlipidemia. EZ exerts its function by inhibiting the intestinal absorption of biliary and dietary cholesterol. EZ treatments restore basal cholesterol, triglyceride, and lipid levels [4,6]. Liver histological studies show that treatment with EZ makes it possible to eliminate ballooning, inflammation, and other cellular alterations [1,7].

EZ can be classified as a Class II drug in the Biopharmaceutical Classification System for its poor water-solubility and high permeability [8]. The low oral bioavailability of EZ (35%) is related to its poor gastrointestinal solubility, rapid hepatic first-pass effect, and P-glycoprotein (P-gp) efflux [4,6].

Therefore, there is considerable interest in developing strategies to improve the dissolution rate of EZ and possibly increasing its bioavailability. Different methods have been applied to reduce the crystallinity of EZ, such as nanocrystals [9] and solid dispersions [10,11,12]. Different surfactants such as sodium dodecyl sulphate, polyoxyethylene sorbitan monooleate (Tween^®^ 80), and castor oil polyoxyethylene (Kolliphor^®^ RH40, formerly known as Cremophor^®^ RH40) may be useful as excipients to inhibit the function of the efflux transporter P-gp and, therefore, it will increase the intestinal absorption of EZ [4,6,13]. The emergence of non-conventional techniques such as self-micellizing solid dispersions is of great importance since they could overcome the stability disadvantages of conventional emulsions or micellar systems [3,14]. 

In recent years, our research group has carried out self-micellizing work with different surfactants and carriers such as microcrystalline cellulose (MC) and croscarmellose, which increase dissolution profiles and enhance the wetting of poorly soluble drugs [15,16,17].

Therefore, the aim of this work was to obtain and characterize new EZ self-micellizing solid dispersions. The low crystallinity and the rapid formation of micelles in the intestinal environment could increase the EZ dissolution profiles and its solubility, which could favor drug absorption. Finally, formulations were selected to evaluate their efficacy in lipid parameters and their liver histology in an animal model fed on a high-fat diet versus those fed ezetimibe raw material (EZ-RM).

## 2. Materials and Methods

### 2.1. Materials

Ezetimibe (EZ) was obtained from the Normon Pharmaceutical Co., Ltd. (Madrid, Spain). Croscarmellose sodium and microcrystalline cellulose (Avicel^®^ pH 101) were procured from FMC Corporation (Philadelphia, PA, USA). Kolliphor^®^ RH40 was obtained from Basf Chemical Company (Barcelona, Spain). Water was obtained from Milli-Q water purification system (Billerica, MA, USA). All reagents and chemicals used were of analytical grade.

### 2.2. Methods

#### 2.2.1. Preparation of Formulations

##### EZ Raw Material (EZ-RM) and Physical Mixture (PM)

EZ-RM was used as a reference for characterization (SEM, XPRD, and DSC) and dissolution studies. For the physical mixture (PM), 200 mg of EZ and 500 mg of croscarmellose hydrophilic vehicle (1:2.5, *w*/*w*) were weighed and mixed in a ceramic bowl using a polymeric spatula. Then, 2 g of microcrystalline cellulose diluent was mixed in. The final product was screened to isolate the 0.297–0.840 mm fraction. 

##### Self-Micellizing Solid Dispersion with Kolliphor^®^ RH40 (MS-K)

Self-micellizing solid dispersion systems were prepared containing EZ:Kolliphor^®^ RH40 ratios of MS-K (1:0.1), MS-K (1:0.3), MS-K (1:0.6), and MS-K (1:0.75) (*w*/*w*). Then, 200 mg of EZ were added to 1000 µL of ethanol solution with different proportions of surfactants and dissolved by vortex (Fisherbrand^TM^; Milan, Italy) at 2500 rpm for 2 min. The EZ solution was mixed (in a ceramic bowl using a polymeric spatula) with 500 mg of croscarmellose as a hydrophilic carrier, dried at 40 °C for 24 h, and then sieved (0.840 mm). The microcrystalline cellulose diluent was then mixed to obtain the different formulations. The final product was screened to isolate the 0.297–0.840 mm fraction.

#### 2.2.2. Scanning Electron Microscopy (SEM): Particle Morphology, Size, and Shape

Samples were mounted on a double-faced adhesive tape and sputtered with a thin gold-palladium layer using a sputter coater Emitech K550X (Quorum Technologies; Lewes, United Kingdom). After coating, the samples were analyzed with a Jeol JSM-6400^®^ scanning electron microscope (Jeol Ltd.; Peabody, MA, USA) operated at an acceleration voltage of 20 kV. All micrographs were the product of secondary electron imaging used for surface morphology identification at a magnification of 5000×.

#### 2.2.3. X-Ray Powder Diffraction (XRPD): Structure and Crystal Size Characterization

The XRPD patterns were recorded on an X-ray diffractometer Philips X’Pert-MPD (Malvern Panalytical; Malvern, United Kingdom). This X-ray diffraction measurement was performed by the XRD technological research facility CAI (Centro de Asistencia a la Investigación, Universidad Complutense de Madrid, Spain). The samples were irradiated with monochromatized CuKα radiation (λ = 1.542 Å) and analyzed between 5 and 40° (2θ). The 5°–40° 2θ degree range was scanned at a step size of 0.04° and at 1 s per step in all cases. The voltage and current used were 30 kV and 30 mA, respectively.

#### 2.2.4. Differential Scanning Calorimetry (DSC)

DSC thermograms were obtained using an automatic thermal analyzer system TC 15, TA controller (Mettler^®^ Toledo; Toledo, OH, USA). Temperature was calibrated using Indium Calibration Reference Standard (transition point: 156.60 °C). Samples were accurately weighed into aluminum pans, then hermetically sealed with aluminum lids and heated from 25 °C to 250 °C at a heating rate of 10 °C/min under constant purging of dry nitrogen at 20 mL/min. An empty pan sealed in the same way as the sample was used as a reference.

#### 2.2.5. Dissolution and Solubility Studies

Dissolution studies were performed using the USP paddle method (apparatus 2) in ERWEKA DT 80 (ERWEKA GmbH; Langen, Germany) dissolution equipment. The apparatus was set with a rotational speed of 50 rpm and 500 mL of dissolution medium containing 0.45% of sodium lauryl sulphate in 0.05 M sodium acetate buffer, adjusted to pH 4.5 (USP42-MF37, 2019). The temperature was maintained at 37.0 ± 0.5 °C throughout the dissolution study. Different formulations with an amount equivalent to 10 mg of EZ were placed in the dissolution vessels. Samples were collected periodically and filtered through a 0.45 μm filter (Acrodisc^®^, Port Washington, NY, USA). The quantity of EZ was determined at 233 nm using a UV-VIS spectrophotometer (Jasco^®^ Analitica S.L.; Madrid, Spain). The cumulative amount of EZ released from the system was determined from the following calibration curve *y* = 0.0389*x* (µg/mL) – 0.0051 (*r*^2^ = 0.9993) across the range 1–15 µg/mL. This method was validated according to ICH Q2 (R1) (CPMP/ICH/381/95). Each determination at each time was performed in triplicate and the error bars on the graphs represent the standard deviation.

Solubility studies were performed using an excess amount of each of the self-micellar systems that were added in test tubes containing 3 mL acetate buffer, 0.05 M (pH 4.5). After vortexing, the tubes were subjected to a water bath with shaking at 37 °C for five days. Samples were filtered through a 0.45 μm filter (Acrodisc^®^, Port Washington, NY, USA). The amount of EZ was determined at 233 nm using the method described in the dissolution studies.

#### 2.2.6. Animal Study

Twenty-four male Wistar rats weighing between 200 and 250 g were supplied from Envigo Rms Co., Ltd. (Barcelona, Spain). The study was carried out in the Animal Experimentation Centre at the University of Alcalá de Henares following the Ethical Committee Regulations of the University, Community of Madrid PROEX 041/18, project identification code ES280050001165 (27 April 2018). The animals were housed in standard cages in 12 h light-dark cycles and had unrestricted access to food and water throughout the experiment. The animals in the study were fed on a high-fat diet (HFD) composed of fats and cholesterol (18 g of fat and 2 g of cholesterol per 100 g of diet) that was administered for eight weeks.

Experiments were performed on four groups of animals (*n* = 6). At the end of the eight weeks of feeding with a HFD, the serum lipid profile was measured for the induction of hyperlipidemia. Treatments of EZ-RM and MS-K formulation (1:0.75), equivalent to a 3 mg/kg dose of EZ, were suspended in sodium carboxymethylcellulose (0.75% *w*/*v*) and 0.4 mL of the treatments were administered to the rats through oral gavage. The hyperlipidemic control group received only 0.4 mL of sodium carboxymethylcellulose (0.75% *w*/*v*). After four weeks of treatment, the animals were anesthetized and sacrificed by cardiac puncture to collect the blood of each animal.

#### 2.2.7. Lipid Profile Analysis

Before extracting the samples, the animals underwent fasting for 12 h to avoid interference with the analyses. The blood samples were introduced in tubes. Serum was separated by centrifugation at 4000 rpm for 20 min (M12P centrifuge; Neuation Technologies, Gandhinagar, India). For all groups, the lipid profiles for total cholesterol (TC), triglycerides (TG), low-density lipoprotein (LDL), and high-density lipoprotein (HDL) were estimated using commercial diagnostic kits (Biosystems Barcelona, Spain and Spinreact Gerona, Spain) and the data were represented as mg/dl. Values are reported as mean ± S.D. The effect in the rat model of hypercholesterolemia was evaluated by one-way ANOVA test using Statgraphic (Statgraphic Technologies, The Plains, VA, USA) followed by Tukey’s test.

#### 2.2.8. Histopathological Analysis

A 5 mm thick section of liver tissue was cut from the center of each hepatic lobe and fixed in 40 g/L buffered formaldehyde, processed by standard techniques, and embedded in paraffin. The sections were stained with hematoxylin-eosin. Steatosis was assessed by a morphological semiquantitative approach and graded as follows: 1 (mild = 5–30% of hepatocytes affected), 2 (moderate = 30–60%), and 3 (severe >60%) [5]. The specimens were also examined for the histological features of Mallory bodies, ballooning degeneration, acidophilic necrosis, sinusoidal fibrosis, and polymorph nuclear infiltrates. The histological evaluation of the liver sections was performed by an experimental pathologist.

## 3. Results and Discussion

### 3.1. SEM Characterization

**Figure 1:**
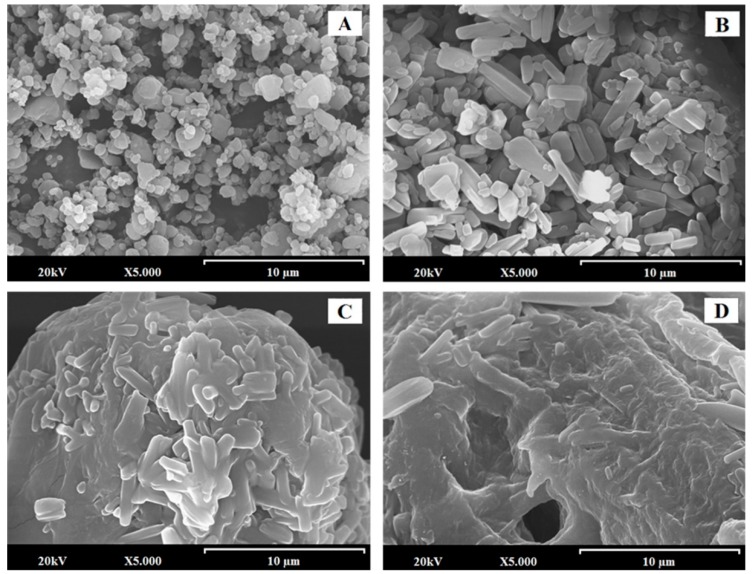
SEM micrographs of surface-modified ezetimibe formulations: ezetimibe raw material (EZ-RM) (**A**). Physical mixture (PM) (**B**). MS-K (1:0.3) (**C**), and MS-K (1:0.75) (**D**). Photographs were taken at a magnification of 5000×.

EZ-RM (Figure 1A) presented small particles with spherical shape and crystals with sizes of 0.5–1.5 µm. This morphology is common in nano/microparticles of EZ [9,18]. PM at low magnification (200×) shows a long fiber structure (50–100 µm approx.), corresponding to the carrier (croscarmellose/microcrystalline cellulose) with small prismatic crystal (orthorhombic) sizes of EZ (0.5–1.5 µm) on its surface (data not shown). At higher magnifications of 5000×, PM (Figure 1B) shows the aggregation of EZ crystals on the surface of the croscarmellose/microcrystalline cellulose fibers. This crystals are identified as EZ by energy dispersive X-ray microanalysis (SEM-EDX) [19,20].

Self-micellar systems have shown significant changes in the surfaces of the transporter particles. Thus, on the surface of MS-K (1:0.3) (Figure 1C), it is possible to identify the prismatic crystals of EZ that are partially covered by a homogeneous surfactant film. Finally, the highest proportions of surfactants in the MS-K self-micellar system (1:0.75) (Figure 1D) produced a thicker homogeneous film on the surface with prismatic forms of EZ trapped inside. Different studies show that high proportions of surfactant ease the wettability and dispersion in the dissolution medium [17,21].

### 3.2. X-Ray Powder Diffraction (XRPD): Structure and Crystal Size Characterization

**Figure 2:**
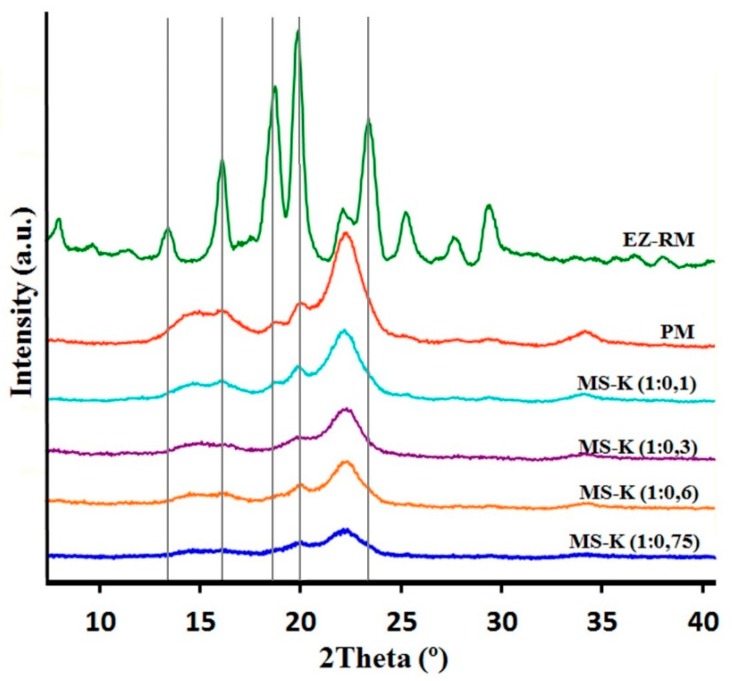
XRPD patterns of ezetimibe raw material (EZ-RM), physical mixture (PM), MS-K (1:0.1), MS-K (1:0.3), MS-K (1:0.6), and MS-K (1:0.75).

X-ray diffraction patterns of EZ raw material (EZ-RM), physical mixture (PM), and micellar systems (MS-K 1:0.1, MS-K 1:0.3, MS-K 1:0.6, and MS-K 1:0.75) are exhibited in Figure 2.

The PXRD of EZ-RM shows sharp peaks at diffraction angles (2θ) of 13.62,16.06, 18.63, 20.16, and 23.59°. This crystal structure was described as anhidrous EZ by different authors [22]. PXRD patterns of PM formulation show two important halos between 13 and 18° and 18 and 25° 2θ. These halos were attributed to a higher crystallinity of the croscarmellose carrier and the semicrystalline nature of microcrystalline cellulose (MC) [17]. In the PM it is possible to observe on this semicrystalline halo the presence of the most representative peaks of EZ with diffraction angles at 18.63 and 20.16° 2θ. 

The presence of the non-ionic surfactant (Kolliphor^®^ RH40) produces an important change in the diffraction parameters. The presence of low ratios of surfactant MS-K (1:0.1) show a similar intensity of the most representative peaks of the EZ (18.63 and 20.16° 2θ) compared to the PM. Furthermore, MS-K (1:0.3) shows a greater decrease in the crystallinity of the EZ (18.63 and 20.16° 2θ) and in the two semicrystalline halos in comparison with the PM and the MS-K (1:0.1). Possibly, MS-K (1:0.3) ratios are required to achieve decreases in the crystallinity of the croscarmellose carrier produced during the drying process. Figure 2 shows that the use of high amounts of surfactant (MS-K 1:0.6 and MS-K 1:0.75) allows for a mostly amorphous structure of EZ. In addition, these proportions of surfactant are necessary to produce a significant decrease in the intensity of the semicrystalline halos of croscarmellose and MC. This result indicates that at these ratios, the surfactant is able to inhibit the crystallization process produced in the croscarmellose carrier during the self-micellizing solid dispersions process. Previous works indicated that the use of surfactants such as Tween 80 or Soluplus^®^ decreased the crystallinity of EZ or produced amorphous forms of EZ [12,18,23].

### 3.3. Differential Scanning Calorimetry (DSC)

**Figure 3:**
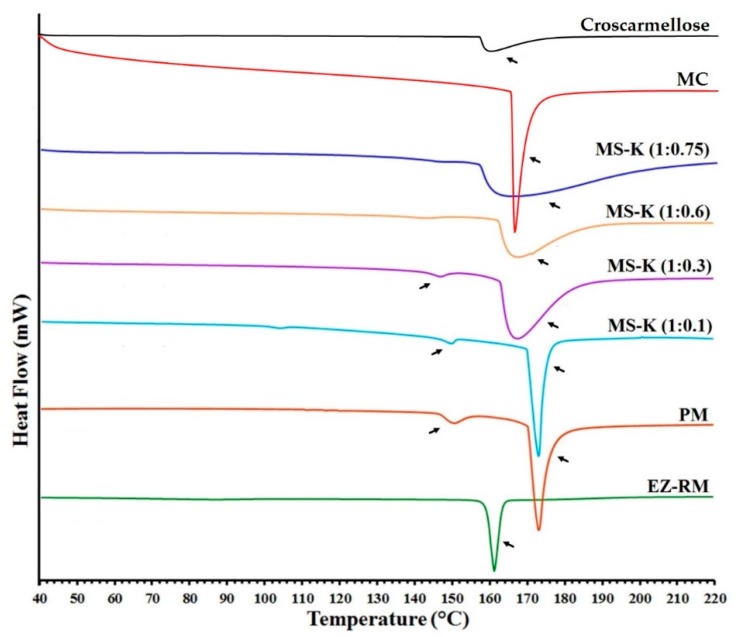
DSC thermograms of croscarmellose, microcrystalline cellulose (MC), ezetimibe raw material (EZ-RM), physical mixture (PM), MS-K (1:0.1), MS-K (1:0.3), MS-K (1:0.6), and MS-K (1:0.75).

Figure 3 shows the DSC curves of EZ raw material (EZ-RM), physical mixture (PM), and the micellar systems MS-K (1:0.1), MS-K (1:0.3), MS-K (1:0.6), and MS-K (1:0.75). EZ-RM presented a sharp endothermic peak at 162.24 °C and an enthalpy that matches a crystalline substance (77.02 J/g), while croscarmellose (159.37 °C) and microcrystalline cellulose (MC) (163.66 °C) showed broad endothermic peaks that coincide with semicrystalline substances. The PM displayed a first endothermic peak at 150.34 °C corresponding to the croscarmellose carrier and a second broad endothermic peak at 173.17 °C corresponding to EZ–MC interaction attributed to the inclusion of EZ molecules within the endothermic peak of the MC. The increase in the enthalpy value was attributed to the greater crystallinity of the MC during the granulation and drying process.

MS-K (1:0.1) shows a slight displacement in the endothermic peaks of croscarmellose (148.56 °C) and the inclusion of the EZ within the MC (172.79 °C). The lower enthalpy of fusion of croscarmellose indicates a decrease in its crystallinity. At the same time, the increase in the enthalpy of fusion of the second peak could be related to the inclusion of the EZ within the MC carrier and the partial recrystallization of this excipient during the self-micellization process. However, higher EZ: Kolliphor^®^ RH40 ratio (MS-K 1:0.3) resulted in decreases in crystallinity in croscarmellose (147.50 °C) and EZ-MC interaction (167.53 °C) (Figure 3). The presence of higher surfactant ratios (MS-K 1:0.3) may decrease the enthalpy of fusion of the drug and carrier and thus facilitate the amorphous forms of EZ and reduce the recrystallization of the MC carrier. These results confirm the increase in crystallization of the MC carrier observed in the PXRD studies for PM and MS-K (1:0.1), and the decrease in crystallinity of the croscarmellose carrier for MS-K (1:0.3). Similar processes of partial amorphization of EZ have been used to obtain EZ nanoparticles [24] or micro/nanoparticles of poorly soluble drugs [25,26]. However, the inclusion of MS-K (1:0.6) and MS-K (1:0.75) surfactant ratios does not display the endothermic peak attributed to croscarmellose (Figure 3). These surfactant proportions allow amorphous forms to be obtained in poorly soluble drugs [14,17]. Both formulations (MS-K 1:0.6 and MS-K 1:0.75) showed a significant decrease in the second endothermic peak. These results confirm that the EZ in MS-K (1:0.6) and MSK-K (1:0.75) had an amorphous form, while the MC carrier had a semicrystalline halo attributed to the process of inhibiting its recrystallisation. These results match with the amorphous form of EZ observed in the PXRD and support the presence of a film on the carrier surface in SEM studies. This surfactant film and the decrease in croscarmellose crystallization could improve the wettability of these micellar systems and decrease the EZ aggregation process during dissolution studies [4,27].

### 3.4. In Vitro Drug Release

**Figure 4:**
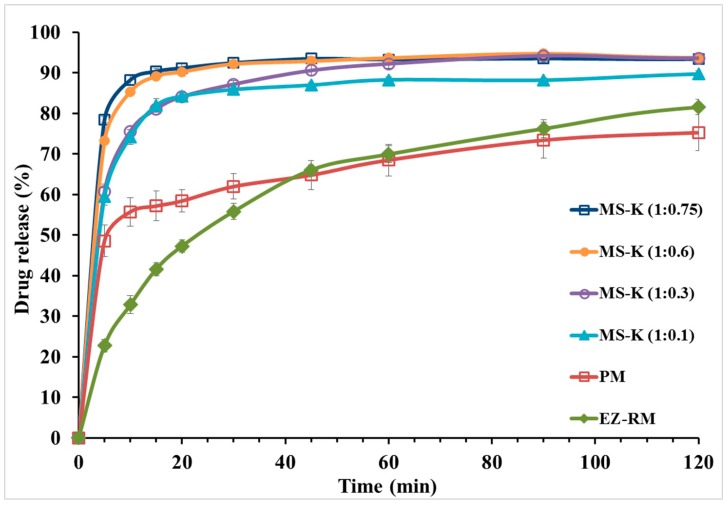
In vitro dissolution profiles of ezetimibe raw material (EZ-RM), physical mixture (PM), MS-K (1:0.1), MS-K (1:0.3), MS-K (1:0.6), and MS-K (1:0.75).

Figure 4 shows the dissolution profiles of EZ-RM, physical mixture (PM), and the micellar systems MS-K (1:0.1), MS-K (1:0.3), MS-K (1:0.6), and MS-K (1:0.75). EZ-RM shows a slow solubility profile (32.79 ± 2.20% at 10 min and 66.03 ± 1.17% at 45 min). The solubility profile of EZ-RM is similar to those reported by other authors [4,7]. PM shows only a slight improvement in its dissolution profile with increases of 1.70-fold at 10 min compared to EZ-RM. Under these conditions, the slight increase in the dissolution profile was attributed to the improved wettability due to the hydrophilic superdisintegrant included in the PM. Different technological processes with hydrophilic excipients have been applied to prevent the aggregation process in hydrophobic drugs such as EZ [9,23].

The micellar system in the solid state with different proportions of surfactant produced a significant increase in the initial dissolution profiles (Figure 4). After 10 min, the dissolved percentages for MS-K (1:0.1) showed a 2.26-fold increase compared to EZ-RM. At 45 min, the percentage of dissolution of MS-K (1:0.1) increased compared to the PM (86.95 ± 0.64% and 64.76 ± 3.58%, respectively). The initial dissolution profiles for MS-K (1:0.1) at 10 min could be related to a decrease in EZ crystallinity and an improvement in wettability due to the inclusion of EZ in the croscarmellose hydrophilic vehicle observed in PXRD and DSC studies [9,12]. 

MS-K (1:0.3) showed a fast dissolution rate. Thus, there was a difference between dissolved percentages of MS-K (1:0.3) and EZ-RM (75.55 ± 2.20% and 32.79 ± 0.48%, respectively) at 10 min. At 45 min, MS-K (1:0.3) showed an improvement in the dissolution properties (90.54 ± 1.24%) compared to MS-K (1:0.1) (86.95 ± 0.64%). This increase of dissolved drug from MS-K (1:0.3) could be due to the production of a micellar structure that allows to enhance the drug dissolution profile. Previous studies showed that with low amounts of surfactant and pH < 6.0 (gastrointestinal medium) the number of micelles decreased [17]. However, micellar systems with high ratios of surfactant, MS-K (1:0.6) and MS-K (1:0.75), presented increased dissolution rates from the initial times (85.27 ± 0.13% and 88.21 ± 1.33% at 10 min, respectively). Moreover, at 45 min, MS-K (1:0.6) and MS-K (1:0.75) also had dissolution percentages close to 90% (92.83 ± 0.85% and 93.45 ± 0.48%, respectively). The micellar systems MS-K (1:0.6) and MS-K (1:0.75) had the highest dissolution percentages at 10 min. These results may be related to the presence of surfactant on the surface of the EZ particles (see SEM studies), which may reduce the aggregation of hydrophobic EZ particles during dissolution studies [23]. 

Finally, although no significant differences (*p* > 0.05) were observed between MS-K (1:0.60) and MS-K (1:0.75) in the procedures dissolved at 10 and 45 min, the solubility studies showed a significant increase (*p* < 0.05) at acetate buffer 0.05 M (pH 4.5), exhibiting concentrations of 18.03 ± 0.34 µg/mL for MS-K (1:0.6) and 26.90 ± 0.49 µg/mL for MS-K (1:0.75). Previous studies have proven that with a pH < 6.0, the formation of micelles can be reduced [28]. However, the higher surfactant ratio for MS-K (1:0.75) could improve the formation of micelles at pH 4.5. These characteristics led us to select the micellar system MS-K (1:0.75) for efficacy studies.

### 3.5. Evaluation of the Efficacy of EZ Formulations

#### 3.5.1. Total Lipid Profile

**Figure 5:**
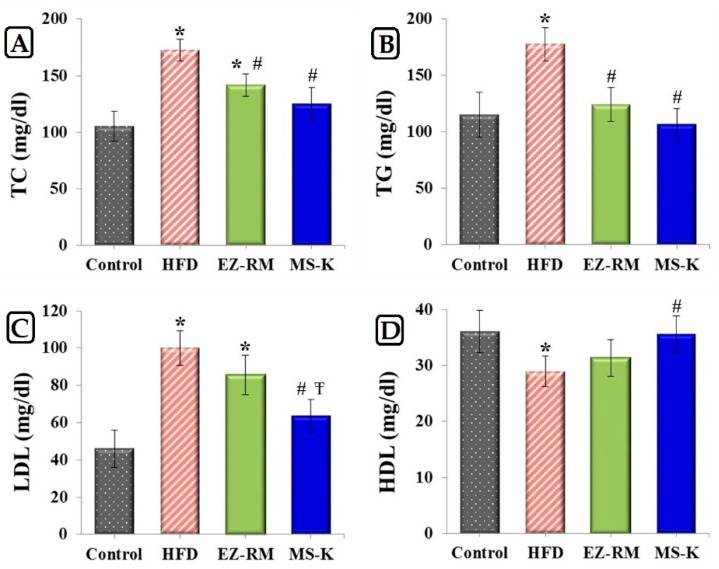
Serum levels of (**A**) total cholesterol (TC), (**B**) triglycerides (TG), (**C**) low-density lipoproteins (LDL) and (**D**) high-density lipoproteins (HDL) in studied group after 4 weeks of treatment. Control group, high-fat diet (HFD) group, ezetimibe raw material (EZ-RM) and MS-K (1:0.75). Treatments equivalent to 3 mg/Kg of EZ. Results are expressed as the means ± SD, *n* = 6 per group, * *p* < 0.05 vs. control group, # *p* < 0.05 vs. HFD group, Ŧ *p* < 0.05 vs. EZ-RM group.

Figure 5 shows the levels of TC, TG, LDL, and HDL after four weeks of treatment for the control group, HFD group, EZ-RM, and MS-K (1:0.75). The HFD group data show significantly elevated levels (*p* < 0.05) of 1.64-, 1.54-, and 2.17-fold for TC, TG, and LDL, respectively, and a reduced level (*p* < 0.05) of 0.80-fold for HDL values compared to the control group. According to these results, our high-fat diet was adequate to induce a hyperlipidemic effect in vivo. These cholesterol values were similar to those obtained by other authors using similar high-fat diets with cholesterol [22].

The EZ-RM group displays an improvement in the serum lipid profile (Figure 5). This group shows a significant decrease (*p* < 0.05) of 0.82- and 0.70-fold for TC and TG values, respectively, compared to HFD group. As illustrated in Figure 5, there was no significant difference (*p* > 0.05) in the serum levels of LDL and HDL between the EZ-RM and HFD groups. A similar antihyperlipidemic effect was obtained in other studies with EZ HP-β-CD inclusion complexes and commercial tablets compared with EZ nanoemulsions [8]. At the end of the treatment, the EZ-RM group showed higher TC values (1.33-fold) than in the control group. In this study, the treatment group MS-K (1:0.75) showed the best lipid-lowering effect. Thus, the TC, TG, and LDL levels for the micellar formulation showed a significant decrease (*p* < 0.05) of 0.74-, 0.60- and 0.64-fold, respectively, and an increase of 1.23 times (*p* < 0.05) in the HDL level compared to the HFD group (Figure 5). This enhanced protection against hyperlipidemia of the MS-K (1:0.75) group compared to the EZ-RM group was attributed to the faster oral absorption of EZ in the micellar formulation than in the EZ-RM one. The fast dissolution rate of MS-K (1:0.75) observed in the dissolution studies plays an important role in the increased intestinal absorption of the EZ [4]. Various studies have indicated that the presence of different surfactants in EZ systems could alter the permeability of the membrane and inhibit the P-gp efflux of EZ in the intestine in vivo [6,9]. Future in vivo pharmacokinetic studies of EZ formulations with different surfactants should be done to evaluate the influence of each surfactant in improving the intestinal absorption of EZ.

#### 3.5.2. Histopathological Study

**Figure 6:**
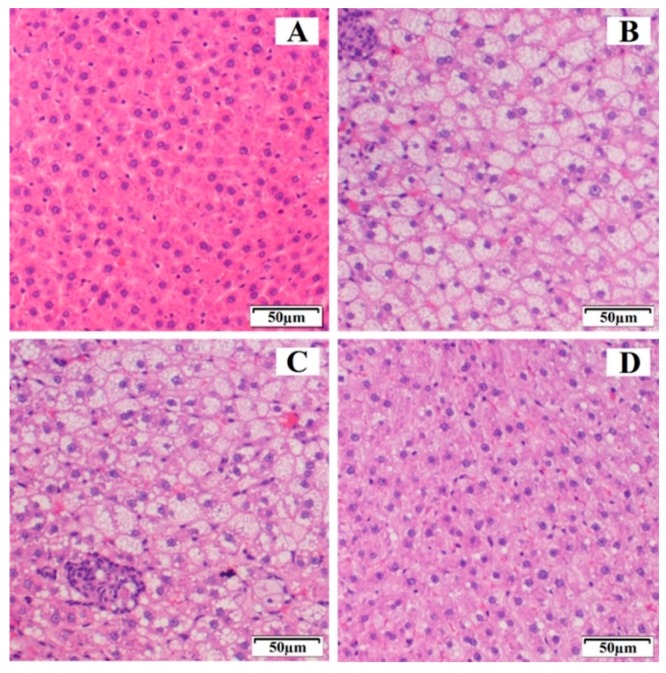
Histological representation (hematoxylin and eosin 20×) of degrees of hepatic steatosis in Wistar rats after 4 weeks of treatment. (**A**) Control group, (**B**) HFD group, (**C**) ezetimibe raw material (EZ-RM), and (**D**) MS-K (1:0.75).

Figure 6 shows the histopathological examination of the control group, HFD, EZ-RM, and MS-K (1:0.75) groups after four weeks of treatment. The control group (Figure 6A) displayed normal liver tissue without steatosis, inflammation, or ballooning. The HFD group (Figure 6B) had a high score (score 3) according to the scale of Marin V. et al. [5], and is therefore related to a severe degree of steatosis. In addition to the histopathological examination, the HFD group presented different diet-induced pathological abnormalities such as intracytoplasmic fat vacuolization in most hepatocytes, inflammatory cells, and granulomas. These histological findings matched the data obtained from the biochemical analysis and indicate that an excessive deposit of lipids and the accumulation of cholesterol and triglycerides in the liver led to hepatic steatosis [3].

The EZ-RM group (Figure 6C) showed severe steatosis (score 3), similar to the high-fat diet group, although the tissue of the animals in this group had a slightly improved histological appearance compared to the HFD group, with a similar fat and granuloma vacuolization but a lower reaction of the inflammatory cells. Interestingly, the histological findings of EZ-RM coincided with high data values of TC and LDL compared to the control group at the end of the treatment. High biochemical data during treatment with EZ-RM could explain the hepatic steatosis observed in this group. This result suggests that a longer treatment is necessary to reduce the liver steatosis observed during the histological examination of the liver sample [29].

An improvement was observed after treatment with EZ-MS (1:0.75) (Figure 6D). The histopathological examination of the liver samples showed hepatic steatosis with inflammatory cells, although less affected areas of hepatocytes could also be observed. These studies revealed a decrease in the scores for hepatic steatosis, which is reduced to moderate (score 2) compared to the HFD and EZ-RM groups. The absence of abnormalities such as areas of inflammatory cells or granulomas coincided with an improvement in TC, TG, LDL, and HDL levels in the MS-K (1:0.75) group. The normalization of lipid profiles may be related to an inhibited progression of liver steatosis in the MS-K (1:0.75) group. The histological findings and the previous results of our study confirmed the superior performance of MS-K (1:0.75) compared to EZ-RM, suggesting that an improvement in the physicochemical characteristics of EZ could enhance its therapeutic efficiency. Liver histological studies show that treatment with EZ is able to eliminate lobular inflammation and achieve an improvement in severe hepatic steatosis [1,7].

## 4. Conclusions

This study clearly demonstrates that the addition of Kolliphor^®^ to ezetimibe in solid dispersions with croscarmellose as a carrier creates micellar systems with rapid dissolution profiles. 

SEM studies proved that micellar systems with higher surfactant ratios showed a larger film layer of surfactant on the carrier surface and entrapped the EZ within the croscarmellose carrier. The PXRD and DSC studies of MS-K (1:0.6) and MS-K (1:0.75) showed that the surfactant created an amorphous form compared to EZ-RM. These results show a significant decrease in crystallinity when the proportion of Kolliphor^®^ was increased.

Dissolution studies confirmed that high surfactant ratios in the MS-K (1:0.6) and MS-K (1:0.75) micellar systems improve their dissolution profiles compared to EZ-RM. At maximum absorption pH (pH 4.5), formulation with higher ratios of surfactant MS-K (1:0.75) showed a significant increase (*p* < 0.05) in their solubility values. 

Biochemical data in hyperlipidemic rats (HFD) revealed that the treatments with the micellar systems containing the highest surfactant ratios (MS-K 1:0.75) had similar levels of TC, TG, LDL, and HDL to those obtained in the control group.

A greater decrease in liver steatosis was found after treatment with MS-K (1:0.75) compared to EZ-RM. Probably, the presence of TC and lipid levels similar to the control group for longer periods could explain the improvement in liver steatosis observed in the histopathological studies.
